# Stable isotopes ( ^15^N) facilitate non‐invasive labelling of large quantities of macroinvertebrates across different species and feeding types

**DOI:** 10.1002/ece3.11539

**Published:** 2024-06-18

**Authors:** Julian Enss, Milen Nachev, Maik A. Jochmann, Torsten C. Schmidt, Christian K. Feld

**Affiliations:** ^1^ Aquatic Ecology, Faculty of Biology University of Duisburg‐Essen Essen Germany; ^2^ Centre for Water and Environmental Research University of Duisburg‐Essen Essen Germany; ^3^ Instrumental Analytical Chemistry, Faculty of Chemistry University of Duisburg‐Essen Essen Germany

**Keywords:** ^15^NH_4_Cl, dispersal measurement, isotopic enrichment, lowland stream, phytobenthos, POM

## Abstract

While macroinvertebrate dispersal operates at the individual level, predictions of their dispersal capabilities often rely on indirect proxies rather than direct measurements. To gain insight into the dispersal of individual specimens, it is crucial to mark (label) and capture individuals. Isotopic enrichment with ^15^N is a non‐invasive method with the potential of labelling large quantities of macroinvertebrates. While the analysis of ^15^N is widely utilised in food web studies, knowledge on the specific utility of isotopic enrichment with ^15^N for mass labelling of macroinvertebrate individuals across different taxa and feeding types is limited. Previous studies have focused on single species and feeding types, leaving gaps in our understanding of the broader applicability of this method. Therefore, this study aimed to test and compare isotopic mass enrichment across several macroinvertebrate taxa and feeding types. We released ^15^NH_4_Cl at five stream reaches in North‐Rhine Westphalia, Germany, and successfully enriched 12 distinct macroinvertebrate taxa (Crustacea and Insecta). Significant enrichment was achieved in active and passive filter feeders, grazers, shredders and predators, and predominantly showed positive correlations with the enrichment of the taxa's main food sources phytobenthos and particulate organic matter. Enrichment levels rose rapidly and peaked at distances between 50 m and 300 m downstream of the isotopic inlet; significant enrichment occurred up to 2000 m downstream of the isotopic inlet in all feeding types. Macroinvertebrate density estimates on the stream bottom averaged to a total of approximately 3.4 million labelled individuals of the 12 investigated taxa, thus showing the high potential of isotopic (^15^N) enrichment as a non‐invasive method applicable for mass labelling across different macroinvertebrate feeding types. Hence, isotopic enrichment can greatly assist the analysis of macroinvertebrate dispersal through mark‐and‐recapture experiments, as it allows to measure the movement at the level of individual specimens.

## Graphical abstract

Isotopic enrichment offers a non‐invasive approach to label macroinvertebrates and contributes to our understanding of dispersal dynamics in aquatic ecosystems. By releasing ^15^NH_4_Cl into stream reaches, we successfully labelled 3 million individuals of 12 macroinvertebrate taxa and observed significant enrichment up to 2000 m downstream, demonstrating the method's general applicability for labelling large amounts of macroinvertebrates.

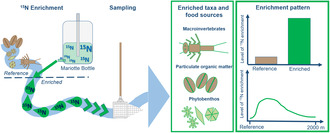

## 1 INTRODUCTION

Dispersal is a fundamental ecological process involving the movement of individuals between discrete habitat patches (Bilton et al., 2001). It is a crucial factor in the life cycle of aquatic macroinvertebrates as it ensures the gene flow between metapopulations (Briers et al., 2004) and the (re‐)colonisation of habitats after the species' release from natural and anthropogenic stressors (Brooks & Boulton, 1991; Vos et al., 2023; Winking et al., 2016), and compensates for riverine downstream drift in lotic systems (Hershey et al., 1993). Macroinvertebrate dispersal is driven by a wide range of abiotic and biotic drivers, for example, by stream flow velocity (James et al., 2008; Naman et al., 2017; Schülting et al., 2016), the presence of predators (Hernandez & Peckarsky, 2014; Lancaster, 1990) and the infestation of macroinvertebrates with parasites (Prati et al., 2023; Vance, 1996) that varies by habitat and therefore may affect different life stages.

Mathematical modelling can help understand the complex relationships that are involved in dispersal and thus facilitate the prediction of dispersal and its underlying processes. Several studies illustrate the utility of dispersal modelling by combining theoretical considerations on dispersal with empirically observed patterns of field studies (Peredo Arce et al., 2021; Radinger et al., 2014; Sondermann et al., 2017). Modelling can help to reconstruct dispersal patterns observed in past studies as well as anticipate probabilities of future dispersal (Sondermann et al., 2017). In order to make sufficiently precise and reliable statements, however, modelling requires a comprehensive understanding of the distances that the individuals of a taxon's population disperse during the taxon's life‐cycle stages and lifetimes until reproduction. Dispersal distances could be derived from field measurements (Kovats et al., 1996; Malicky, 1987) or, as an alternative, from species‐specific dispersal traits (Li et al., 2016, 2018; Sarremejane et al., 2020). However, a widely acknowledged criticism against the use of species‐specific distances or species trait‐based proxies of dispersal in predictive dispersal models is that dispersal operates at the level of individual specimens rather than at the level of a species’ entire population (Doerr & Doerr, 2005; Driscoll et al., 2014; Tonkin et al., 2018). Therefore, for accurate predictions, knowledge about the distances travelled by individual members of a population and the proportion of the population engaging in dispersal is essential (Lancaster & Downes, 2017; Sondermann et al., 2017).

Previous research indicates that dispersal distances follow leptokurtic distributions, where only a small fraction of individuals engage in extensive dispersal, while the majority of individuals within a population exhibit a limited mobility (Petersen et al., 1999; Radinger et al., 2014). Studies suggest that macroinvertebrate dispersal distances follow a leptokurtic distribution too (Nathan et al., 2012). However, the knowledge of individual dispersal distances travelled by macroinvertebrates remains limited, as does our understanding of the proportion of stationary and mobile individuals within macroinvertebrate populations. To address these knowledge gaps and to assess both dispersal distances and the mobile fractions of macroinvertebrate populations, labelling of individuals is necessary. A promising non‐invasive labelling technique reported to be capable of labelling large quantities of stream macroinvertebrates is the use of stable isotopes (Briers et al., 2002). Stable isotopes of biogenic elements naturally occur as a composition of heavy and lightweight isotopes, with the heavier isotopes (e.g. ^2^H, ^18^O, ^13^C, ^15^N) typically exhibiting a much lower abundance in nature. The ratio of ^14^N to ^15^N in air, for example, is 99.634: 0.366 (Coplen et al., 2002). By releasing biologically available ^15^N into the environment, this ratio can be artificially altered in autotrophs (e.g. benthic algae) and their consumers (e.g. macroinvertebrates), thereby changing their isotopic compositions.

While ^15^N is widely studied in aquatic ecosystems to investigate the uptake, turnover and retention processes of nitrogen as well as the trophic interactions in aquatic food webs (Sánchez‐Carrillo & Álvarez‐Cobelas, 2018; Tank et al., 2000), the knowledge of its utility for mass labelling of large quantities of macroinvertebrates in order to assess their dispersal distances is limited; several studies confirmed the general feasibility of isotopic enrichment of stream macroinvertebrates with ^15^N, but largely focused on single species that feed as grazers (Hershey et al., 1993) or gathering collectors (Briers et al., 2004; Caudill, 2003; Macneale et al., 2005). However, little is known about the feasibility of isotopic labelling across different macroinvertebrate taxa and feeding types, that is, covering grazers, shredders, gathering collectors, active and passive filter feeders and predators. If applicable, this labelling technique would allow to label large quantities of populations of numerous species and hence support the investigation of macroinvertebrate dispersal at the level of individual specimens for whole communities.

Here, we present the results of a study that aimed to investigate the feasibility of isotopic enrichment with ^15^N across several stream macroinvertebrate taxa of different feeding types. We hypothesised that besides ‘grazers’ (Hershey et al., 1993) and ‘detritus feeder’ (= gathering collectors) (Briers et al., 2004; Caudill, 2003; Macneale et al., 2005), mass enrichment is feasible also for other feeding types (H1). Shredders feed on particulate organic matter (POM, e.g. fallen leaves) and thereby also ingest the biofilm of fungi and algae that grows on POM and facilitates its decomposition (Bastias et al., 2020). It is likely that the highly productive biofilm incorporates sufficient ^15^N to also significantly label their consumers. Likewise, active and passive filter feeders would get enriched, as they also feed on POM and suspended particles. Predators would get enriched, when feeding on enriched prey organisms. The enrichment of macroinvertebrates irrespective of their feeding behaviour should allow to label extremely high numbers—even millions—of macroinvertebrate individuals (Briers et al., 2004).

Furthermore, we hypothesised that ^15^N enrichment levels vary across the food chain due to differences in the enrichment of main food sources (H2). In particular, grazers are expected to exhibit a high level of enrichment as they directly feed upon phytobenthos (i.e. benthic algae), which is known to be a productive sink of nitrogen. In contrast, shredders and gathering collectors would become less strongly enriched, because their food primarily consists of dead organic matter. As to the longitudinal pattern of isotopic enrichment in the stream continuum, we hypothesised a pronounced enrichment directly downstream of the isotope inlet, that is, the point where ^15^N is released into the water. Enrichment would peak close to the inlet and from there subsequently decline until the ^15^N signal would reach natural background levels (Hershey et al., 1993) (H3). This pattern reflects the expectation of rapid incorporation of ^15^N into primary producers near the source, resulting in a substantial rise, followed by a decrease in the ^15^N enrichment signal with increasing distance from the isotope inlet.

## 2 MATERIALS AND METHODS

### 2.1 Study area

**FIGURE 1:**
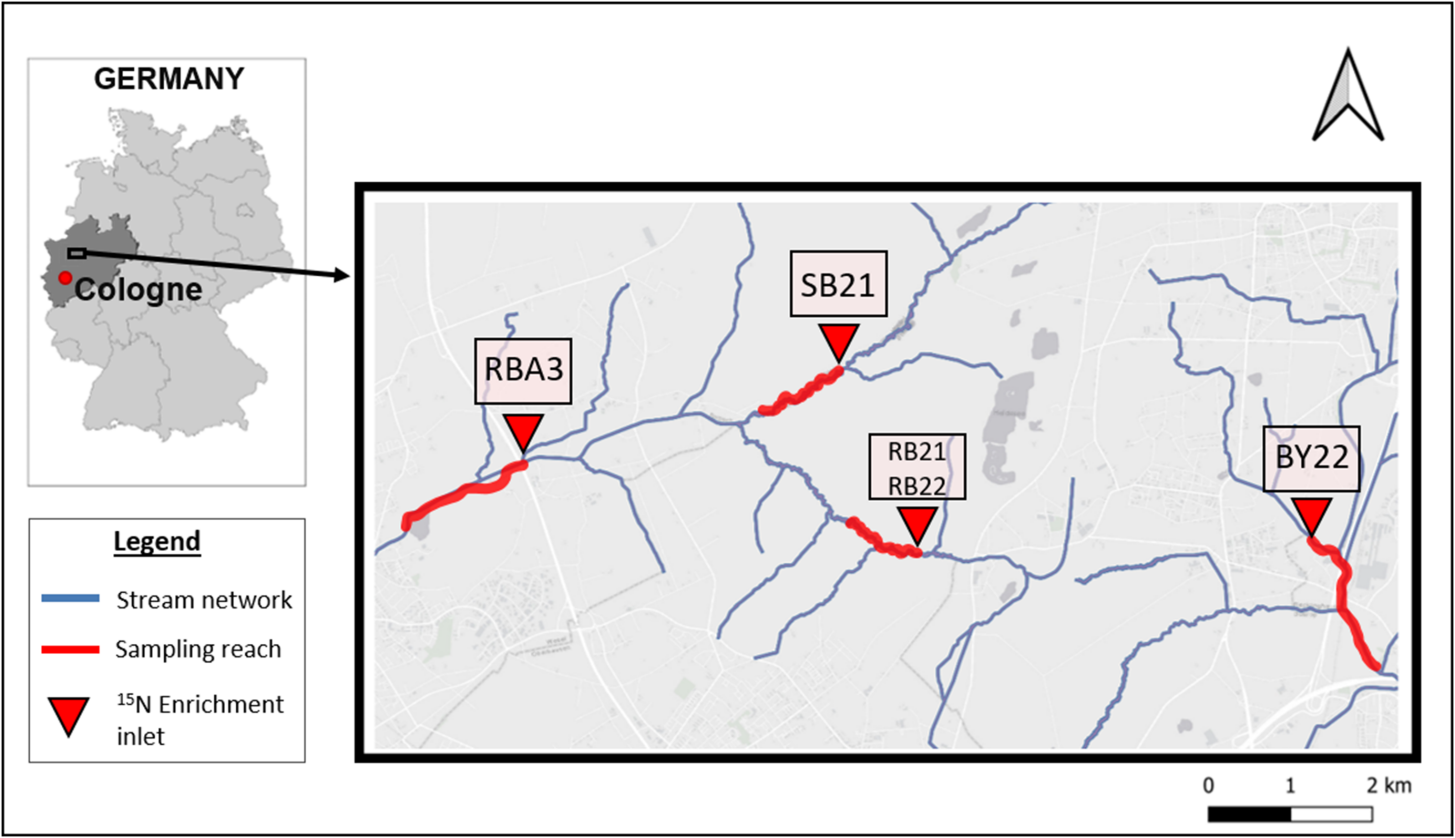
Location of 2 km sampling reaches (thick red lines) in the Rotbach (RB, SB) and the Boye (BY) catchments in North‐Rhine Westphalia, Germany. Visual differences in sampling reaches are attributed to the pronounced curvature (with meanders) of the reaches RB21/RB22 and SB21.

The study was carried out at five sampling reaches (each 2 km in length) in two sand‐bottom lowland streams (Rotbach/Schwarzbach and Boye) in North Rhine‐Westphalia, Germany (Figure 1). The Rotbach (incl. its major tributary Schwarzbach) has a rural catchment with a near‐natural and mainly forested upstream section (reach RB21/RB22 and SB21) that is also characterised by near‐natural hydromorphological conditions. Since reaches RB21 and RB22 are spatially identical but differ in the sampled year, they are considered as separate samples. Further downstream, extensive agriculture (horse meadows) dominates the land use adjacent to the stream course, which is hydromorphologically degraded. Several hydromorphological restorations have been implemented in the past decade, which aimed to restore the stream course, its bed and bank conditions and the riparian corridor. Study reach RBA3 is located within a restored section. The upper catchment of the Boye is dominated by intensive agriculture (meadows and arable land). Here, the stream is hydromorphologically altered and has to be pumped against a gradient due to mining subsidence as a result of intensive mining in the catchment area. Reach BY22 is located downstream of a pumping station within a straightened section accompanied by a narrow forested riparian corridor. The mean discharges at the sampling reaches are given in Table 1.

**TABLE 1:** Stable isotope release (^15^NH_4_Cl) and discharge conditions during the enrichment period at five sampling reaches in the Boye (BY), Schwarzbach (SB) and Rotbach (RB). Mean values were calculated from up to 6 weekly measurements within the enrichment period (42 days).

Site	^15^NH_4_Cl (g)	Mean discharge (±SD) (L s^−1^)	Mean dilution (mg/L s^−1^)	Enrichment period
BY22	20	650 ± 324	31	03/31/2022–05/12/2022
RB21	20	415 ± 349	48	05/11/2021–06/21/2021
RB22	20	433 ± 146	46	03/29/2022–05/10/2022
RBA3	30	1841 ± 855	16	03/29/2022–05/10/2022
SB21	40	300 ± 143	133	08/17/2021–09/28/2021

### 2.2 Biological sampling

#### 2.2.1 Population density estimation

Biological sampling took place in 2021 and 2022. For population density assessment, a representative section (100 m in length) was selected within each sampling reach and subdivided into five subsections (each 20 m in length). Macroinvertebrates were sampled in three of the five subsections (upstream end, middle and downstream end) using a hand net (shovel‐sampler, frame size: 25 × 25 cm, mesh size: 500 μm) in line with the German standard methodology (Meier et al., 2006). In each subsection, the width of the watercourse was measured, and the different bed substrates were sampled according to their estimated proportions of the total area of the stream bed, with a maximum of 11 samples per subsection. Sampling within each subsection proceeded against the flow, that is, starting from the downstream margin and moving upwards, while the movement followed a zig‐zag line to ensure that the sampling covered the habitat variability of frequent substrates in the subsection. To take an individual sample, the hand net was placed directly on the stream bed and the area of 25 × 25 cm directly upstream of the net was disturbed by hand at a depth of 5 cm.

**FIGURE 2:**
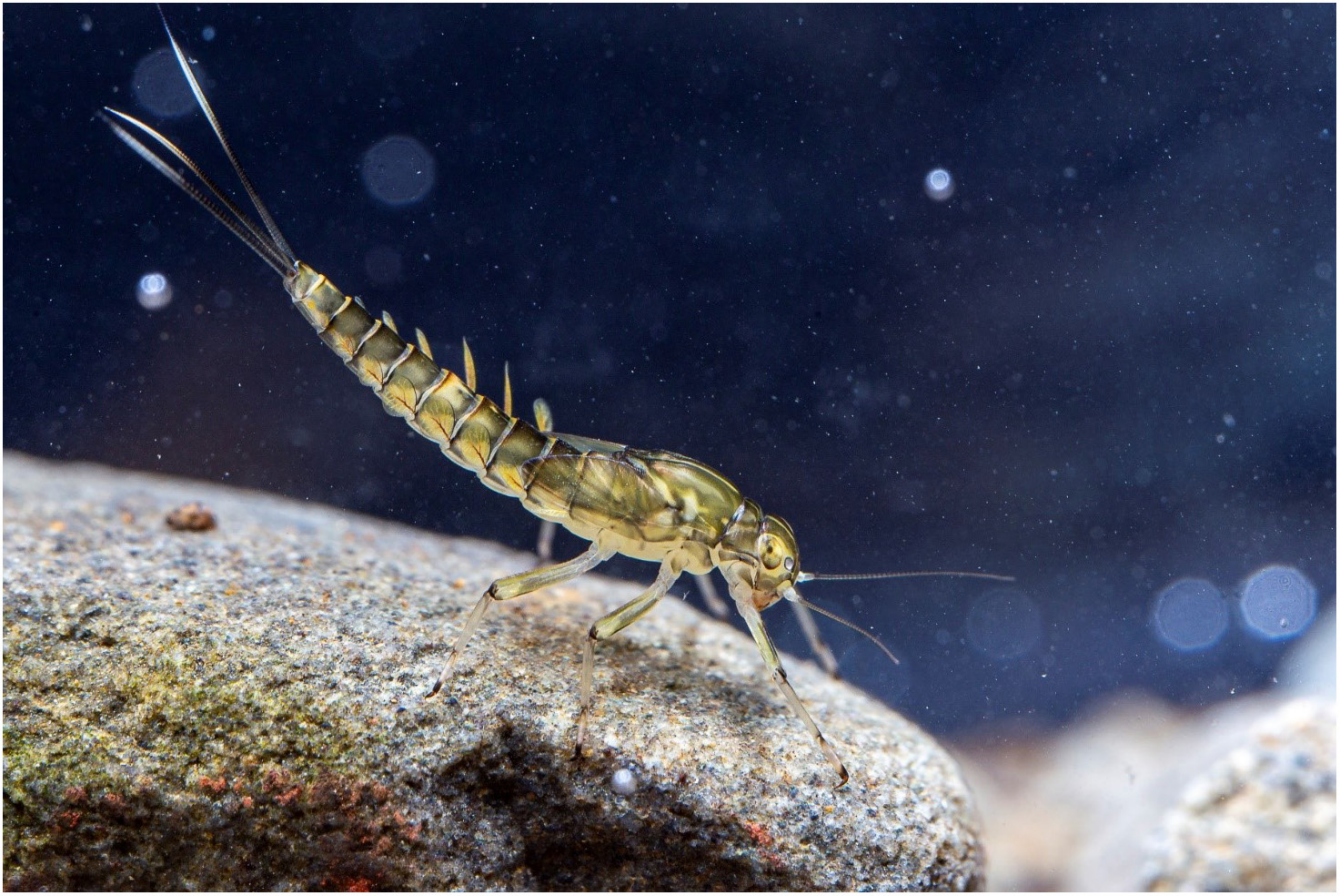
Larva of *Baetis* sp., Leach 1815 (Insecta: Ephemeroptera). Approximately 800,000 individuals of this grazing taxon were successfully labelled with ^15^N during this enrichment experiment (Picture: Julian Enss).

Based on availability, abundance and consideration of different feeding types, altogether 12 model taxa were selected for the labelling experiment (Table 2, Figure 2). These model taxa were fixed in 96% ethanol for transport and storage until processing in the laboratory. To estimate the population density of model taxa in a reach, the mean density (±1 SD) within the three sampled 20 m subsections was calculated and divided by 20 to obtain the taxon's density (and its variability) per 1 m of stream length. Because reach RB21 and RB22 are spatially identical and differ only in the sampled year, the population density estimates from 2021 were also used for the experiment in 2022.

**TABLE 2:** Enrichment (*δ* ^15^N) of investigated taxa and two food sources at nine distances downstream of the isotope inlet.

Class/order	Taxon	Feeding type	*N*	Reference	Distance downstream to isotope inlet (m)								
					50 m	100 m	200 m	300 m	500 m	750 m	1000 m	1500 m	2000 m
				**Mean *δ* ^15^N (‰)**									
Crustacea: Amphipoda	*Gammarus pulex* (Linnaeus, 1758)	Shredder	336	4.8 ± 1.3	**44.8 ± 47.1**	**32.5 ± 33.9**	**33.5 ± 30.4**	**30.8 ± 28.2**	**17.7 ± 17.7**	**17.2 ± 15.8**	**13.4 ± 10.8**	**10.1 ± 7.6**	7.3 ± 4.3
Insecta: Ephemeroptera	*Ephemera danica* Muller, 1764	Active filter feeder	271	5.7 ± 1.1	**42.7 ± 35.0**	**34.7 ± 29.6**	**28.8 ± 31.4**	**24.4 ± 20.1**	**31.6 ± 32.6**	**32.2 ± 36.4**	**25.9 ± 24.3**	**16.1 ± 16.0**	**11.3 ± 16.8**
	*Baetis* sp. Leach, 1815	Grazer	169	5.4 ± 1.5	**64.3 ± 72.4**	**59.7 ± 68.5**	**59.5 ± 54.6**	**83.4 ± 76.8**	**66.4 ± 38.5**	**100.2 ± 75.0**	**76.3 ± 50.9**	**16.7 ± 6.2**	**34.6 ± 22.0**
Insecta: Megaloptera	*Sialis* sp. Latreille, 1802	Predator	65	7.5 ± 0.8	8.1 ± 1.0	**9.4 ± 3.4**	7.5 ± 2.0	**12.9 ± 7.1**	**10.2 ± 5.3**	**14.1 ± 2.2**	**23.2 ± 20.2**	7.0 ± 2.0	7.0 ± 1.7
Insecta: Plecoptera	*Nemoura* sp. Latreille, 1796	Shredder	78	4.4 ± 0.8	**109.0 ± 44.0**	**104.4 ± 38.6**	**98.3 ± 18.2**	**115.9 ± 24.2**	**95 ± 40.9**	**69.5 ± 20.8**	**67.3 ± 18.7**	**38.2 ± 7.6**	**25.1 ± 2.7**
Insecta: Trichoptera	*Hydropsyche angustipennis* (Curtis, 1834)	Passive filter feeder	83	5.2 ± 0.9	**49.3 ± 39.5**	**45.4 ± 27.5**	**42.0 ± 17.9**	**55.9 ± 16.9**	**48.9 ± 19.3**	**57.5 ± 18.8**	**39.2 ± 23.5**	**7.2**	**14.9 ± 3.6**
	*Polycentropus irroratus* Curtis, 1835	Predator	22	7.2 ± 0.5		**80.3 ± 7.0**	**81.1**	**76.3 ± 47.2**			**56.3 ± 20.9**	**33.8 ± 9.3**	**17.7 ± 1.2**
	*Chaetopteryx villosa* (Fabricius, 1798)	Shredder	31	6.3 ± 0.1	**13.0 ± 13.9**	**36.2 ± 30.7**	**32.5**	**10.9 ± 3.8**	**10.2 ± 2.4**		**9.1 ± 2.9**	**9.2 ± 6.5**	7.9 ± 2.4
	*Halesus radiatus* (Curtis, 1834)	Shredder	81	0.9 ± 1.7	**12.0 ± 5.5**	**11.4 ± 5.4**	**7.2 ± 3.5**	**13.1 ± 7.9**	**13.5**	**11.0 ± 8.4**	2.6 ± 1.5	1.0 ± 2.0	0 ± 2.4
	*Limnephilus lunatus* Curtis, 1834	Shredder	53	6.1 ± 0.7	**9.2 ± 3.2**	**17.9 ± 11.5**	**12.8 ± 7.2**	**12.7 ± 5.7**	**12.3 ± 3.5**	**11.4 ± 2.0**	**9.4 ± 3.5**	**9.8 ± 2.7**	**9.1 ± 2.2**
	*Potamophylax cingulatus* (Stephens, 1837)	Shredder	14	1.5 ± 0.9			**27.4**		**14.6**	**19.9 ± 7.0**	**11.2 ± 1.3**	**4.8 ± 1.6**	
	*Potamophylax rotundipennis* (Brauer, 1857)	Shredder	24	1.1 ± 0.7		**9.2**	**4.4 ± 2.9**		**19.1 ± 25.9**	**5.2**	**5.6 ± 0.9**	**3.6 ± 0.7**	
**Food source**													
Particulate organic matter (POM)			319	0.5 ± 1.2	2.3 ± 7.3	**4.6** ± **8.5**	2.6 ± 4.6	1.3 ± 4.8	1.9 ± 5.7	2.0 ± 3.7	0.8 ± 2.8	0.6 ± 2.4	1.1 ± 3.9
Phytobenthos (PB)			327	4.5 ± 2.2	**16.6** ± **14.9**	**22.8** ± **17.0**	**14.8** ± **12.7**	8.1 ± 8.1	**11.1** ± **12.7**	7.7 ± 5.2	7.5 ± 6.4	6.8 ± 6.1	5.9 ± 4.4

*Note*: Feeding types reflect the main assignment acc. to freshwaterecology.info (Schmidt‐Kloiber & Hering, 2015). Reference measurements were derived from samples taken 50 m upstream of the isotope inlet. *N*, number of specimens analysed for each taxon. Bold values indicate enrichment levels above Reference +2 SD (Macneale et al., 2004). Empty lines indicate that a species has not been detected at this distance.

#### 2.2.2 Biological sampling of labelled specimens and food sources

After enrichment, model taxa, phytobenthos (PB) and particulate organic matter (POM) were sampled at nine sampling sites below the release point (50, 100, 200, 300, 500, 750, 1000, 1500 and 2000 m) and a non‐labelled site located 50 m above the release point (reference) to evaluate the enrichment with and depletion of ^15^N in the biomass over a distance of up to 2000 m downstream of the release point (biological sampling).

Up to 10 individuals of the model taxa were sampled using a hand net (frame size: 25 × 25 cm, mesh size: 500 μm) at each sampling site (see ‘Section 2.2.1’).

Phytobenthos was brushed into labelled containers with a toothbrush from at least five different stones or pieces of dead wood. POM was collected by hand near the banks as well as in the middle of the streams. All biological samples were stored in labelled containers containing 96% ethanol at −20 °C until stable isotope analysis.

### 2.3 Isotopic enrichment

Enrichment with heavy nitrogen (^15^N) was accomplished by using isotopically enriched ^15^NH_4_Cl (Silantes, minimum 99 atom. % ^15^N purity), which was diluted in 40 L distilled water for each enrichment experiment. This tracer solution was released through a Mariotte's bottle, which assured a consistent release of the solution independent of the hydrostatic pressure within the bottle. The solution was evenly released during the 42‐day enrichment period, which was equivalent to a release rate of approx. 39.7 mL per hour. In order to calculate the dilution of NH_4_Cl during the enrichment period, weekly discharge measurements were taken using a vane anemometer (MiniAir20; Schiltknecht Messtechnik, Switzerland) (Table 1). At 10 evenly spaced locations along the stream's cross‐section, flow velocity was measured at 20% and 80% depth and averaged to get a mean flow velocity per location. The mean discharge was then calculated from the mean value of all 10 locations multiplied by the cross‐sectional area of the site (Herschy, 2014). A fertilisation effect by nitrogen was not expected due to the small amounts of added ^15^NH_4_Cl (Briers et al., 2004).

### 2.4 Stable isotope analysis

For the stable isotope analysis all sampled specimens of target taxa, phytobenthos and POM were freeze dried (Heto PowerDry LL3000; Thermo Fisher Scientific, Waltham, USA) and homogenised using a stainless steel micro pestle. Each sample was weighed using an electronic precision scale (accurate to 0.001 mg, M2P, Sartorius AG, Göttingen, Germany) (benthic invertebrates in the range: 0.5–1.0 mg; particulate organic matter and phytobenthos: 2.0–3.0 mg) and folded into 4 × 6 mm tin capsules for solids (IVA Analysentechnik e.K., Meerbusch, Germany) by pressing out the air voids. Samples were prepared as triplicates if the amount of homogenised sample material was sufficient or into duplicates or single samples for taxa with low dry sample mass (*Baetis* sp., *Nemoura* sp.). To avoid contamination, all tools (micro pestle, forceps, stainless steel folding block) used for the handling of samples were previously wiped with acetone (analytical grade, Fisher Chemical, USA). Stable isotopes were analysed using isotope ratio mass spectrometry (IRMS, Isoprime visION, Elementar, Germany) connected to an elemental analyser (EA, Vario ISOTOPE Select, Elementar, Germany) operating in CN‐mode. Acetanilide was used as a laboratory internal standard and was normalised using the international standards USGS40 and USGS41a (both International Atomic Energy Agency, Vienna, Austria). The isotope ratios were calculated and reported in δ‐notation as differences of the isotope ratio of the sample and isotope ratio of an international reference substance (for details see Nachev et al. (2017). *δ* ^15^N for each sample was calculated as the mean *δ* ^15^N of each triplicate, duplicate, or single processed sample.

### 2.5 Data analysis

All statistical analyses and graphical representations of results were conducted using R (v.4.1.2; R Core Team, 2021) in RStudio (v23.6.1; Posit team, 2023). The minimum type‐I error was set to *p* < .05 in all statistical analyses.

In general, a sample (*S*) was considered enriched (=labelled) when its *δ* ^15^N value exceeded the mean *δ* ^15^N + 2SD of the respective reference site upstream of the enrichment inlet *R* (Formula 1) (Macneale et al., 2004, 2005).

(1) $$\text{Enriched}=\bar{x\;}\left(\delta^{15}N_{s}\right)>\bar{x}\left(\delta^{15}N_{R}\right)+2*\sigma_{R}$$

To identify taxon‐specific enrichment (H1), the mean *δ* ^15^N values of the measured individuals of taxon were compared to the respective reference values acc. to Formula 1. If the reference value was missing due to the absence of the specific taxon at the reference site of a reach, the reference value from the closest reach that included the particular taxon was used instead.

The same procedure was applied to determine the enrichment of feeding types and food sources (H2). Therefore, feeding types were assigned to model taxa acc. to Schmidt‐Kloiber and Hering (2015). Taxa with various feeding type assignments (omnivores) were assigned to the main feeding type, that is, the type with the highest individual score.

For each feeding type and food source, an independent samples t‐test was performed to assess whether there were significant differences in mean *δ* ^15^N values between the reference site and sites downstream to the enrichment inlet (function: t.test()).

Pairwise correlations for *δ* ^15^N of phytobenthos and POM with different feeding types at each sampled distance downstream to the ^15^NH_4_Cl enrichment inlet over all sampling reaches were tested for statistical significance using Spearman correlation analysis (function: cor.test()).

To investigate the enrichment along the stream continuum downstream (H3), all sampled individuals of model species and all phytobenthos and POM at each of the nine distances downstream of the enrichment inlet were compared to the respective reference value acc. to Formula 1. This facilitated the determination of the length of the enriched section for each taxon and food source, enabling to observe patterns of enrichment over this specific section.

For each feeding type and food source, an independent samples *t*‐test was performed to assess whether there were significant differences in mean *δ* ^15^N values between the 50–300 m and the 500–2000 m sampling sites downstream to the enrichment inlet in each reach (function: t.test()).

Further, the section length of observable isotopic enrichment was used to estimate the overall number of enriched specimens of each model taxon by multiplying the enriched section length with the taxon‐specific density estimates (see above).

## 3 RESULTS

### 3.1 Taxa density

The taxon‐specific density estimates varied notably between reaches, ranging from 1.1 (*Sialis* sp., BY22) to 362.8 (*Gammarus pulex*, RB21/RB22) individuals per metre of reach length, with densities being generally higher in the predominantly near‐natural forested upstream sections (reach RB21/RB22 and SB21) as compared to the hydromorphologically degraded downstream sections (reach RBA3 and BY22) (Table 3). Several taxa showed a heterogenous distribution across reaches (e.g. *Chaetopteryx villosa*: 0–2.41 Ind. m^−1^, *G. pulex*: 17.08–362.83 Ind. m^−1^) and also across the three 20 m‐subsections within a reach (e.g. *Nemoura* sp.: 3.3 ± 1.7 (mean ± SD), *G. pulex*: 362.8 ± 463.2).

**TABLE 3:** Density estimates and number of enriched specimens of investigated taxa within the enriched sections of the five sampling reaches.

Taxon	Site code	Enriched section (m)	Number of individuals m^−1^, Mean (min–max)	Total number of enriched individuals, Mean (min–max)
*Gammarus pulex* (Linnaeus, 1758)	BY22	2000	5.79 (1.8–15.5)	11,580 (3600–31,000)
	RB21	750	2.41 (1.7–5.5)	1205 (850–2750)
	RB22	1000	5.44 (0–16.3)	10,880 (0–32,600)
	RBA3	2000	90.21 (32.6–136)	180,420 (65,200–272,000)
	SB21	2000	25.74 (10.2–38.7)	51,480 (20,400–77,400)
*Ephemera danica* Muller, 1764	BY22	2000	1.35 (0–4)	2025 (0–6000)
	RB21	1500	1.13 (0–3.4)	565 (0–1700)
	RB22	1000	184.72 (35–298.7)	277,080 (52,500–448,050)
	RBA3	2000	362.83 (59.5–896)	272,122.5 (44,625–672,000)
	SB21	2000	325.44 (98–700)	325,440 (98,000–700,000)
*Baetis* sp. Leach, 1815	BY22	2000	14 (0–28)	2800 (0–5600)
	RB22	200	184.72 (35–298.7)	184,720 (35,000–298,700)
	RBA3	2000	362.83 (59.5–896)	362,830 (595,00–896,000)
	SB21	2000	325.44 (98–700)	244,080 (73,500–525,000)
*Sialis* sp. Latreille, 1802	BY22	500	21.43 (9.3–34)	2143 (930–3400)
	RB22	750	21 (0–63)	15,750 (0–47,250)
	RBA3	2000	2.46 (0.9–4.7)	4920 (1800–9400)
	SB21	1000	2.41 (0–5.5)	3615 (0–8250)
*Nemoura* sp. Latreille, 1796	SB21	2000	4.21 (1.9–6)	8420 (3800–12,000)
*Polycentropus irroratus* Curtis, 1835	SB21	2000	17.08 (6.9–28.3)	34,160 (13800–56,600)
*Chaetopteryx villosa* (Fabricius, 1798)	BY22	500	54.5 (0–163.5)	109,000 (0–327,000)
	RBA3	1500	2.98 (0.9–5.1)	5960 (1800–10,200)
*Halesus radiatus* (Curtis, 1834)	RB21	1000	7.89 (0–17.3)	15,780 (0–34,600)
	RB22	750	8.15 (0–19.2)	16,300 (0–38,400)
*Limnephilus lunatus* Curtis, 1834	BY22	2000	348.8 (259.2–403.2)	697,600 (518,400–806,400)
	RBA3	2000	234.07 (63.4–518.4)	468,140 (126,800–1,036,800)
*Potamophylax cingulatus* (Stephens, 1837)	SB21	1500	55.2 (5.8–148.9)	110,400 (11,600–297,800)
*Potamophylax rotundipennis* (Brauer, 1857)	BY22	1500	4.14 (0–12.4)	6210 (0–18,600)
	RB22	100	23.25 (5.8–57.6)	23,250 (5800–57,600)

### 3.2 Isotopic enrichment

A total of 1227 macroinvertebrate individuals from the five sampling reaches were investigated for isotopic enrichment (Table 2). Among them, 141 individuals originated from the reference site upstream of the enrichment inlet, and 1086 individuals were captured downstream of the enrichment inlet. Within the latter group, 903 individuals (83.1%) were enriched, that is, their *δ* ^15^N value was higher than the mean value plus two times the standard deviation of the reference site. An enrichment was detectable across 12 distinct taxa, encompassing representatives from five taxonomic orders and two classes (Table 2). Mean enrichment of taxa ranged between 9.5‰ (with 20 g ^15^NH_4_Cl), 16.9‰ (30 g) and 54.1‰ (40 g), thus exceeding the taxon‐specific enrichment thresholds by an average of 66.1% (20 g), 108.6% (30 g) and 656.4% (40 g) (Table 1).

### 3.3 Enrichment of macroinvertebrate feeding types and food sources

**FIGURE 3:**
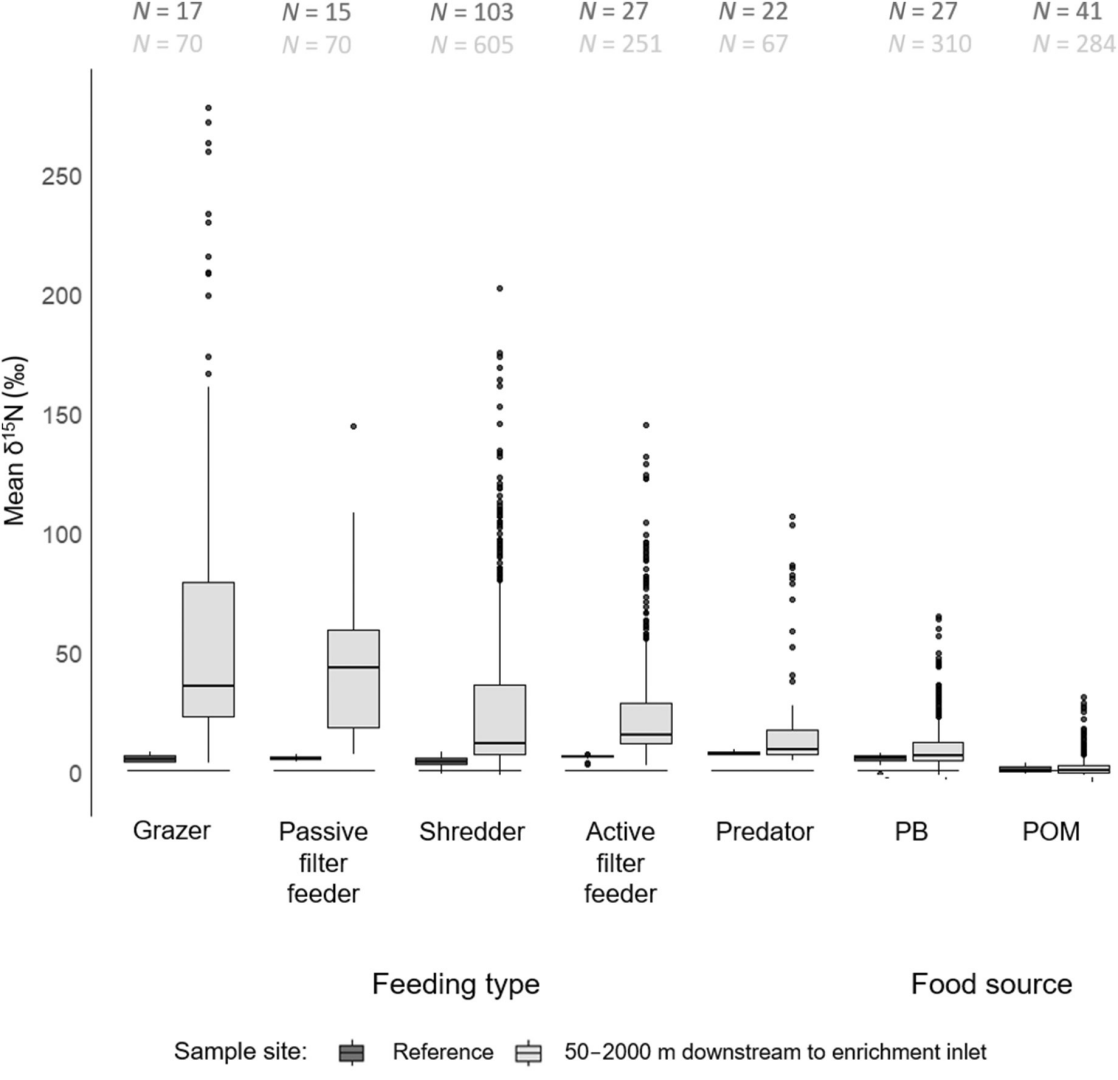
*δ* ^15^N measurements of macroinvertebrate specimens grouped by feeding types and of food sources at non‐enriched reference sites (light grey) and at enriched sites (dark grey). *N*, number of specimens analysed.

The 12 enriched species, categorised into five feeding types (Figure 3), exhibited significant differences in mean *δ* ^15^N values between the reference sites upstream and the sites downstream of the enrichment inlet (*t*‐test, *p* < .001; Figure 3, Table 4). Downstream of the enrichment inlet, grazer displayed the highest mean *δ* ^15^N value (64.61‰ ± 62.44), followed by passive filter feeders (42.70‰ ± 26.74), shredders (28.04‰ ± 35.22), active filter feeders (27.98‰ ± 29.29) and predators (21.62‰ ± 26.73). Notably, PB showed consistently higher mean *δ* ^15^N values (10.79‰ ± 11.26) at all reaches as compared to POM (1.90‰ ± 5.40) downstream of the enrichment inlet. The predominant correlation between feeding types and food sources was positive (Spearman's *ρ* = 0.66, *p* = .04). In general, active filter feeders, predators and shredders exhibited moderate to strong positive relationships (Spearman's *ρ* = 0.429–0.929) with both PB and POM (Table 5). However, reach‐specific differences weakened the overall trend, as correlations were not statistically significant at all reaches for predators and active filter feeders. Notably, although mainly positive correlations were observed, there was one instance of reach‐specific negative correlations of shredders and POM (Reach BY22, Spearman's *ρ* = −0.72, *p* = .02). In general, grazers showed weak and mostly insignificant correlations with both PB and POM. Passive filter feeders occurred at two reaches and showed insignificant correlations with both food sources at one of them, while data were limited at the other reach.

**TABLE 4:** Differences in the mean *δ* ^15^N of macroinvertebrate feeding types and investigated food sources between enriched and non‐enriched reference sites.

Feeding type/food source	Reference		Enriched		*t*	df	*p*
	Mean	SD	Mean	SD			
Active filter feeder	5.7	0.9	28	29	12	254	<.001
Passive filter feeder	5.2	0.9	42	27	12	69.7	<.001
Grazer	5.2	1.5	65	62	12	154	<.001
Shredder	5.2	5.2	28	35	15	701	<.001
Predator	7.4	0.7	22	27	4.4	66.3	<.001
Phytobenthos (PB)	4.7	2.1	11	11	8	215	<.001
Particulate organic matter (POM)	0.5	1.2	1.9	5.4	3.8	273	<.001

*Note*: Significance was tested using individual *t*‐tests.

**TABLE 5:** Pairwise Spearman correlations (*ρ*) of *δ* ^15^N of phytobenthos (PB) and particulate organic matter (POM) with macroinvertebrate specimens of different feeding types.

Food source	Feeding type	Site	*ρ*	*p*	*N*	Significance	Food source	Feeding type	Site	*ρ*	*p*	*N*	Significance
**POM**	Active filter feeder	BY22	−0.393	0.396	7		**PB**	Active filter feeder	BY22	0.000	1.000	7	
		RBA3	−0.224	0.537	10				SB21	0.612	0.066	10	
		**RB21**	**0.648**	**0.049**	**10**	*			RB22	0.624	0.060	10	
		**SB21**	**0.806**	**0.008**	**10**	**			**RBA3**	**0.685**	**0.035**	**10**	*
		**RB22**	**0.818**	**0.007**	**10**	**			**RB21**	**0.758**	**0.016**	**10**	*
	Passive filter feeder	SB21	0.600	0.097	9			Passive filter feeder	SB21	0.367	0.336	9	
	Grazer	RBA3	−0.224	0.537	10			Grazer	SB21	0.100	0.810	9	
		SB21	0.150	0.708	9				BY22	0.257	0.658	6	
		BY22	0.371	0.497	6				**RBA3**	**0.648**	**0.049**	**10**	*
	Shredder	**BY22**	**−0.721**	**0.024**	**10**	*		Shredder	BY22	0.127	0.733	10	
		RBA3	−0.055	0.892	10				RB22	0.406	0.247	10	
		**RB21**	**0.661**	**0.044**	**10**	*			**RBA3**	**0.697**	**0.031**	**10**	*
		**RB22**	**0.818**	**0.007**	**10**	**			**SB21**	**0.830**	**0.006**	**10**	**
		**SB21**	**0.891**	**0.001**	**10**	***			**RB21**	**0.891**	**0.001**	**10**	***
	Predator	RBA3	0.429	0.299	8			Predator	RBA3	0.000	1.000	8	
		**RB21**	**0.833**	**0.015**	**8**				RB21	0.571	0.151	8	
		**SB21**	**0.929**	**0.007**	**7**	**			**SB21**	**0.857**	**0.024**	**7**	*

*Note*: Correlations are based on mean enrichment levels across all sampling reaches and grouped by distance downstream of the isotope inlet. Significant results are indicated in bold; significance levels are indicated as *(*p* < .05), **(*p* < .01) and ***(*p* < .001).

### 3.4 Enrichment distances

**FIGURE 4:**
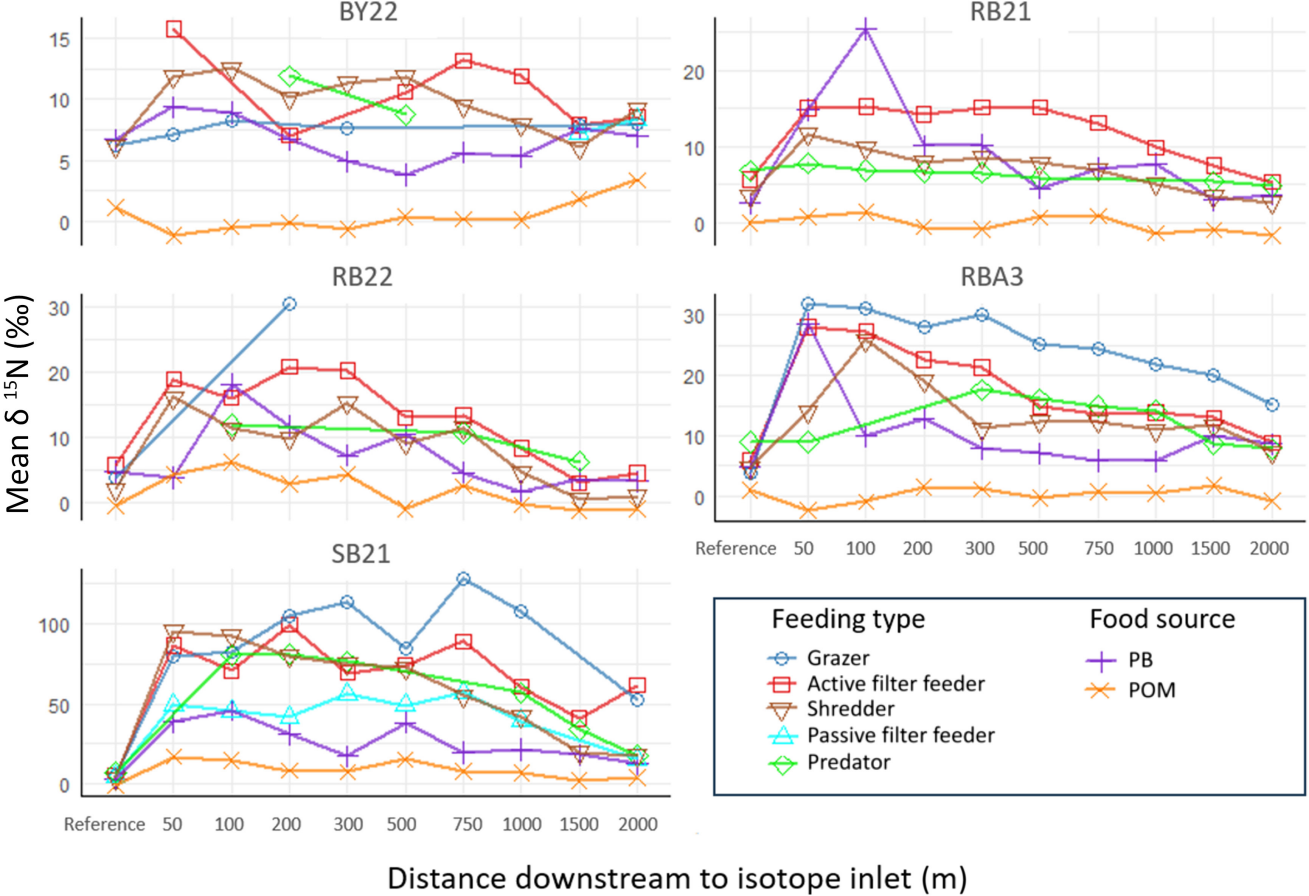
Mean *δ* ^15^N contents of investigated macroinvertebrate specimens (per feeding type) and of food sources along the five sampling reaches. Reference marks the non‐enriched site 50 m upstream of the isotope inlet. (For better clarity, SDs around mean values are not shown).

The mean enrichment for all feeding types and food sources exhibits a sharp increase downstream of the enrichment inlet, peaking at 32.3‰ (50 m) and reaching its highest value at 33.3‰ (100 m). Subsequently, there is a gradual decrease in enrichment, with values of 30.5‰ (200 m), 27.6‰ (300 m), 24.6‰ (500 m) and 25.7‰ (750 m), followed by a more noticeable reduction to 19.1‰ (1000 m) and 9.2‰ (1500 m) until 10.8‰ (2000 m). Over 80% of the enriched taxa and food sources exhibited their highest mean *δ* ^15^N levels within the first 300 m downstream to the enrichment inlet. Differences in mean *δ* ^15^N levels between the sites at 50–300 m and 500–2000 m were significant (*t*‐test, *p* < .001; Table 6) for all reaches, except for BY22. Across all tested feeding types and food sources, successful enrichment was observed up to the maximum sampled distance of 2000 m downstream to the enrichment inlet, with reach‐specific variations to the maximum distances (Figure 4, Table 3).

**TABLE 6:** Differences in the mean *δ* ^15^N of macroinvertebrates and investigated food sources at the sites 50 m–300 m and 500 m–2000 m downstream to the enrichment inlet. Significance was tested using individual *t*‐tests.

Reach	50–300 m		500–2000 m		*t*	df	*p*
	Mean	SD	Mean	SD			
BY22	6.4	5.6	6.5	5.7	−0.1	228.7	0.93
RB21	10.2	7.5	5.8	4.9	6.0	248.9	<0.001
RB22	11.9	8.5	4.6	4.9	8.2	176.7	<0.001
RBA3	15.6	14	8.9	6.7	5.4	202.2	<0.001
SB21	70.9	50	47.7	39.9	5.8	484.1	<0.001

### 3.5 Number of enriched individuals

Extrapolating taxa‐specific densities across all reaches and taking into account the maximum achieved enrichment distance per reach and target taxon, we estimated a mean of approx. 3.4 million individuals (min: 1.1 million, max: 6.7 million) enriched with ^15^N. Taxa densities, enrichment distances and the numbers of enriched individuals notably varied across the reaches. A minimum of at least 565 *Sialis* sp. along an enriched stream length of 500 m (BY22) and up to 697,600 *G. pulex* along an enriched stream length of 2000 m (SB21) were successfully enriched (Table 3). The number of enriched individuals of the same taxon varied greatly between all reaches with the highest differences found in *G. pulex* with 20 times more individuals in SB21 compared to RBA3, while variability was relatively small in *C. villosa* with only four times more enriched individuals in RBA3 as compared to BY22.

## 4 DISCUSSION

### 4.1 Feasibility of isotopic enrichment using ^15^N

This study aimed to investigate the applicability of stable isotopic enrichment (^15^N) to label large amounts of macroinvertebrates irrespective of their taxon identity and feeding behaviour. Enrichment experiments were conducted across five reaches of sand‐bottomed lowland streams in Germany, involving the collection and analysis of macroinvertebrate taxa, as well as two major food sources (phytobenthos and particulate organic matter) for their ^15^N enrichment. In contrast to previous enrichment studies that primarily focussed on single species/taxa (Briers et al., 2004; Caudill, 2003; Hershey et al., 1993; Macneale et al., 2005), this study confirmed the successful isotopic enrichment of 12 macroinvertebrate taxa representing active and passive filter feeders, grazers, shredders and predators. Reliable isotopic enrichment was detected at distances of up to 2 km downstream of the enrichment inlet, which demonstrates the general suitability of ^15^N isotopic enrichment for mass labelling of multiple taxa, that is, communities. The population density estimations suggest that on average more than 3 million individuals belonging to five taxonomic orders (Amphipoda, Ephemeroptera, Plecoptera, Trichoptera and Megaloptera) were successfully labelled, which supports our first hypothesis. Although it is important to acknowledge that these high numbers of individuals represent extrapolated estimates rather than precise measurements, they by far exceed the numbers that are typically achieved by traditional mark‐and‐recapture methods (e.g. Lehmann (1967), Rawer‐Jost et al. (1998). Even under the most conservative scenario based upon the lowest densities of the target taxa observed in our study, more than one million individuals were successfully labelled. This order of magnitude is supported, for example, by Briers et al. (2004), who successfully labelled 1.5 million specimens of the stonefly *Leuctra inermis* with ^15^N. Thereby, we would assume that the actual number of labelled specimens in our study was much higher, as we based our estimates only upon the 12 targeted taxa. Further, labelling with stable isotopes is advantageous over conventional approaches due to its non‐invasiveness. Manual labelling of animals can induce stress or injury, labels may be lost during molting and they may not adhere to merolimnic taxa upon emergence. However, little is known as to the general applicability of the method to investigate the dispersal of aquatic macroinvertebrates of different orders and across different feeding types.

The level of ^15^N enrichment in the investigated specimens varied strongly among reaches and was primarily attributed to the quantity of ^15^NH_4_Cl that was released during the 6‐week enrichment period. This relationship was found to be independent of the discharge and thus of the dilution factor that was evaluated at all investigated reaches during the enrichment period.

Some ^15^N signatures of POM, phytobenthos and macroinvertebrate specimens of enriched sampling sites were lower than those of the reference samples. This seems contradictory, but could be explained by the variation that was introduced by considering the (pooled) average enrichment levels of several distinct taxa as representative values for a single feeding type over a reach length of 2 km. Furthermore, environmental background levels of ^15^N may change due to different land uses adjacent to the investigated 2 km sections (Hall et al., 2009). These potential sources of variation have been reported to influence *δ* ^15^N measurements at a magnitude that is equivalent to one trophic level (i.e. factor 1.6, (Cremona et al., 2010). However, considering the major objectives of our study, these sources of variability are unlikely to confound the labelling of benthic macroinvertebrates at *δ* ^15^N levels that exceed natural background levels by factor 10–20, as observed in this study. Further, a high level of enrichment proves beneficial for maintaining a distinct signal over extended periods following the enrichment phase. After enrichment, the levels of ^15^N in food sources and taxa that consume them return to their regular background values. Since the molting process during emergence is not anticipated to result in a significant loss of the enriched ^15^N (Hershey et al., 1993) and at least one of the sexes of certain merolimnic macroinvertebrates does not feed in the adult stage (Anderson, 2009; Caudill, 2003), the ^15^N enrichment acquired during the larval stage can be retained. However, many merolimnic species must consume terrestrial material in the adult stage (for egg production by females), which is likely to result in a decrease of *δ* ^15^N in their tissue over time. Macneale et al. (2004), for example, reported a decrease of 2‰ in females of *Leuctra ferruginea* (Plecoptera) between emergence and oviposition.

### 4.2 ^15^N enrichment across feeding types

For simplicity, we considered the predominant feeding type of the investigated macroinvertebrate taxa (acc. to Schmidt‐Kloiber and Hering (2015) for the analysis of enrichment levels across feeding types in our study. We acknowledge that the primary consumers (grazers, filter feeders and shredders) do not exclusively feed on a single food source but may switch between food sources and feeding habits during their aquatic life stages. This may have introduced an unaccounted source of variability to our analyses. However, the focus on the main feeding habit was sufficient to test our hypotheses. We found significant differences in the level of ^15^N enrichment between macroinvertebrate feeding types, which confirms our second hypothesis. The highest enrichment levels were found for grazers (e.g. the mayfly *Baetis* sp.) and active filter feeders (e.g. the mayfly *Ephemera danica*), which may relate to the high enrichment of benthic algae that constitute a major food source of *Baetis* sp. (Brown, 1961). However, the low correlation that was found between the enrichment levels in phytobenthos and grazers suggests that this well‐reported relationship between grazers and their (supposed) primary food source was not well reflected by our results. Enrichment levels of both phytobenthos and grazers also showed a high variability within and across study reaches, which may have masked the expected correlation.

In contrast to grazers, modest and positive correlations between feeding types and the investigated food sources were found for active filter feeders, shredders and predators. Active filter feeders and shredders feed primarily on POM but may also feed on phytobenthos that grow on POM. This is supported by the strong and positive correlation between the enrichment levels of both feeding types and investigated food sources at several study reaches. It is also supported by the high enrichment levels of shredders and active filter feeders at several study sites, which were close to those of grazers. If shredders and active filter feeders were primarily feeding on POM, lower enrichment levels of both feeding types should be expected, as enrichment levels of POM were much lower than those of phytobenthos at all study sites (see also Tank et al., 2018).

The enrichment of the single passive filter feeder exhibited no correlation with enrichment levels of phytobenthos and POM in our study, although the investigated specimens of this species were significantly enriched. The consistent and significant enrichment of two predators (*Sialis* sp. and *Polycentropus irroratus*) confirms that isotopic enrichment is feasible across feeding types and trophic levels (Tank et al., 2000).

### 4.3 Longitudinal patterns in the enrichment with ^15^N

Consistent with our third hypothesis, the observed pattern in the enrichment levels along the stream continuum aligns well with the expected rapid incorporation of ^15^N into primary producers near the source of enrichment. In general, the enrichment was detectable already 50 m downstream of the isotope inlet, then rapidly increased up to 300 m downstream, from where enrichment started to decline gradually. Similar patterns have been identified by previous studies (Briers et al., 2004; Hershey et al., 1993; Macneale et al., 2005), although our results show that a significant enrichment of both food sources and targeted taxa can be achieved at distances of up to 2 km below the isotope inlet (and probably further downstream). This exceeds the maximum enrichment distance of 420 m for *Leuctra inermis* reported by Briers et al. (2004) and of 663 m for *L. ferruginea* reported by Macneale et al. (2005) by factor 3–5 and is probably owed to the comparatively high amount and purity of ^15^NH_4_Cl that we released during our study. Hershey et al. (1993) reported the enrichment of *Baetis* sp. up to 2.1 km downstream from the isotope inlet, yet those results were based upon drifting specimens, which renders the comparison with studies on stationary specimens difficult. We are well aware of the possibility that drifting specimens may have influenced our results too. However, the consistent enrichment patterns along the stream continuum that we observed for *Baetis* sp. and other drift‐prone taxa across all study sections suggest that the vast majority of specimens remained relatively stationary. This consistency in the enrichment patterns suggests a minimal impact of drift on our results.

With regard to the overall enrichment distance, our findings suggest a positive correlation between the quantity of released ^15^N and the length of the enriched section. This in particular would be beneficial, if the enrichment of a maximum number of individuals was aimed at, such as in measuring terrestrial dispersion between the upper reaches of watercourses. In such cases, maximising the number of enriched individuals is crucial, as the likelihood of a recapture decreases with distance. In other cases, the enrichment with lower quantities of stable isotopes might be beneficial, for example, if more control over the length of an enriched section was the objective. This may support dispersal measurements from several closely located reaches, that is, the shorter enrichment distances would allow for a finer spatial resolution.

It is beyond the scope of this study to provide precise recommendations as specific quantities of ^15^N are required in order to achieve certain levels of enrichment or certain lengths of enriched sections. Such an attempt might at least suffer from the complex interplay of spatiotemporal, chemical and biological covariates influencing the transport of ^15^N along the stream continuum, its assimilation (retention) by primary producers and its transmission through the aquatic food chains. Nevertheless, future studies can draw guidance from the findings of this research and the studies conducted by Briers et al. (2004), Caudill (2003), Hershey et al. (1993) and Macneale et al. (2005) when determining the quantity of ^15^N required for successful labelling.

## 5 CONCLUSION

^15^N labelling emerges as a powerful tool for mass labelling of macroinvertebrates. The findings derived from our experiment demonstrate the applicability of this method across various feeding types for the labelling of whole communities. Moreover, the exceptionally high number of approximately 3.4 million labelled individuals among 12 targeted taxa emphasises the scalability and efficiency of stable isotope labelling compared to traditional mark‐and‐recapture methods. This non‐invasive technique overcomes practical challenges associated with manual labelling and holds the potential to contribute significantly to the field of dispersal ecology, bridging gaps in knowledge and enhancing our ability to comprehend the complexities of dispersal patterns, while providing valuable data for the development and refinement of dispersal models.

## AUTHOR CONTRIBUTIONS

**Julian Enss:** Formal analysis (lead); investigation (lead); methodology (equal); visualization (lead); writing – original draft (lead). **Milen Nachev:** Methodology (equal); supervision (supporting); writing – review and editing (equal). **Maik A. Jochmann:** Methodology (equal); writing – review and editing (equal). **Torsten C. Schmidt:** Writing – review and editing (equal). **Christian K. Feld:** Conceptualization (lead); methodology (equal); supervision (lead); writing – review and editing (equal).

## CONFLICT OF INTEREST STATEMENT

The authors declare that they have no competing financial interests or personal relationships that could have appeared to influence the work reported in this paper.

## Data Availability

The data that support the findings of this study are openly available in github via https://github.com/enssjulian/Enss_et_al_2024_Ecology_and_Evolution

## References

[ece311539-bib-0001] Anderson, N. H. (2009). Chapter 164 – Megaloptera: Alderflies, Fishflies, Hellgrammites, Dobsonflies. In V. H. Resh & R. T. Cardé (Eds.), Encyclopedia of insects (2nd ed., pp. 620–623). Academic Press Elsevier. https://www.sciencedirect.com/science/article/pii/B9780123741448001739

[ece311539-bib-0002] Bastias, E. , Ribot, M. , Bernal, S. , Sabater, F. , & Martí, E. (2020). Microbial uptake of nitrogen and carbon from the water column by litter‐associated microbes differs among litter species. Limnology and Oceanography, 65(8), 1891–1902. 10.1002/lno.11425

[ece311539-bib-0003] Bilton, D. T. , Freeland, J. R. , & Okamura, B. (2001). Dispersal in freshwater invertebrates. Annual Review of Ecology and Systematics, 32(1), 159–181. 10.1146/annurev.ecolsys.32.081501.114016

[ece311539-bib-0004] Briers, R. A. , Cariss, H. M. , & Gee, J. H. R. (2002). Dispersal of adult stoneflies (Plecoptera) from upland streams draining catchments with contrasting land‐use. Fundamental and Applied Limnology, 155(4), 627–644. 10.1127/archiv-hydrobiol/155/2002/627

[ece311539-bib-0005] Briers, R. A. , Gee, J. H. R. , Cariss, H. M. , & Geoghegan, R. (2004). Inter‐population dispersal by adult stoneflies detected by stable isotope enrichment. Freshwater Biology, 49(4), 425–431. 10.1111/j.1365-2427.2004.01198.x

[ece311539-bib-0006] Brooks, S. S. , & Boulton, A. J. (1991). Recolonization dynamics of benthic macroinvertebrates after artificial and natural disturbances in an Australian temporary stream. Marine and Freshwater Research, 42(3), 295. 10.1071/MF9910295

[ece311539-bib-0007] Brown, D. S. (1961). The food of the larvae of *Chloeon dipterum* L. and *Baetis rhodani* (Pictet) (Insecta, Ephemeroptera). Journal of Animal Ecology, 30(1), 55. 10.2307/2113

[ece311539-bib-0008] Caudill, C. C. (2003). Measuring dispersal in a metapopulation using stable isotope enrichment: High rates of sex‐biased dispersal between patches in a mayfly metapopulation. Oikos, 101(3), 624–630. 10.1034/j.1600-0706.2003.12467.x

[ece311539-bib-0009] Coplen, T. B. , Böhlke, J. K. , de Bièvre, P. , Ding, T. , Holden, N. E. , Hopple, J. A. , Krouse, H. R. , Lamberty, A. , Peiser, H. S. , Revesz, K. , Rieder, S. E. , Rosman, K. J. R. , Roth, E. , Taylor, P. D. P. , Vocke, R. D., Jr. , & Xiao, Y. K. (2002). Isotope‐abundance variations of selected elements (IUPAC technical report). Pure and Applied Chemistry, 74(10), 1987–2017. 10.1351/pac200274101987

[ece311539-bib-0010] Cremona, F. , Planas, D. , & Lucotte, M. (2010). Influence of functional feeding groups and spatiotemporal variables on the δ^15^N signature of littoral macroinvertebrates. Hydrobiologia, 647(1), 51–61. 10.1007/s10750-009-9798-5

[ece311539-bib-0011] Doerr, E. D. , & Doerr, V. A. J. (2005). Dispersal range analysis: Quantifying individual variation in dispersal behaviour. Oecologia, 142(1), 1–10. 10.1007/s00442-004-1707-z 15378345

[ece311539-bib-0012] Driscoll, D. A. , Banks, S. C. , Barton, P. S. , Ikin, K. , Lentini, P. , Lindenmayer, D. B. , Smith, A. L. , Berry, L. E. , Burns, E. L. , Edworthy, A. , Evans, M. J. , Gibson, R. , Heinsohn, R. , Howland, B. , Kay, G. , Munro, N. , Scheele, B. C. , Stirnemann, I. , Stojanovic, D. , … Westgate, M. J. (2014). The trajectory of dispersal research in conservation biology. Systematic review. PLoS One, 9(4), e95053. 10.1371/journal.pone.0095053 24743447 PMC3990620

[ece311539-bib-0013] Hall, R. O. Jr , Tank, J. L. , Sobota, D. J. , Mulholland, P. J. , O'Brien, J. M. , Dodds, W. K. , Webster, J. R. , Valett, H. M. , Poole, G. C. , Peterson, B. J. , Meyer, J. L. , McDowell, W. H. , Johnson, S. L. , Hamilton, S. K. , Grimm, N. B. , Gregory, S. V. , Dahm, C. N. , Cooper, L. W. , Ashkenas, L. R. , … Arango, C. P. (2009). Nitrate removal in stream ecosystems measured by 15N addition experiments: Total uptake. Limnology and Oceanography, 54(3), 653–665. 10.4319/lo.2009.54.3.0653

[ece311539-bib-0014] Hernandez, S. A. , & Peckarsky, B. L. (2014). Do stream mayflies exhibit trade‐offs between food acquisition and predator avoidance behaviors? Freshwater Science, 33(1), 124–133. 10.1086/674360

[ece311539-bib-0015] Herschy, R. W. (2014). Streamflow measurement, Third edition (3rd ed.). CRC Press.

[ece311539-bib-0016] Hershey, A. E. , Pastor, J. , Peterson, B. J. , & Kling, G. W. (1993). Stable isotopes resolve the drift paradox for Baetis mayflies in an Arctic River. Ecology, 74(8), 2315–2325. 10.2307/1939584

[ece311539-bib-0017] James, A. B. W. , Dewson, Z. S. , & Death, R. G. (2008). The effect of experimental flow reductions on macroinvertebrate drift in natural and streamside channels. River Research and Applications, 24(1), 22–35. 10.1002/rra.1052

[ece311539-bib-0018] Kovats, Z. , Ciborowski, J. A. N. , & Corkum, L. (1996). Inland dispersal of adult aquatic insects. Freshwater Biology, 36(2), 265–276. 10.1046/j.1365-2427.1996.00087.x

[ece311539-bib-0019] Lancaster, J. (1990). Predation and drift of lotic macroinvertebrates during colonization. Oecologia, 85(1), 48–56. 10.1007/BF00317342 28310954

[ece311539-bib-0020] Lancaster, J. , & Downes, B. J. (2017). Dispersal traits may reflect dispersal distances, but dispersers may not connect populations demographically. Oecologia, 184(1), 171–182. 10.1007/s00442-017-3856-x 28349200

[ece311539-bib-0021] Lehmann, U. (1967). Drift und populationsdynamik von Gammarus pulex fossarum koch. Zeitschrift fr Morphologie Und Kologie der Tiere, 60(1–3), 227–274. 10.1007/BF00403495

[ece311539-bib-0022] Li, F. , Sundermann, A. , Stoll, S. , & Haase, P. (2016). A newly developed dispersal metric indicates the succession of benthic invertebrates in restored rivers. The Science of the Total Environment, 569‐570, 1570–1578. 10.1016/j.scitotenv.2016.06.251 27443455

[ece311539-bib-0023] Li, F. , Tonkin, J. D. , & Haase, P. (2018). Dispersal capacity and broad‐scale landscape structure shape benthic invertebrate communities along stream networks. Limnologica, 71, 68–74. 10.1016/j.limno.2018.06.003

[ece311539-bib-0024] Macneale, K. H. , Peckarsky, B. L. , & Likens, G. E. (2004). Contradictory results from different methods for measuring direction of insect flight. Freshwater Biology, 49(10), 1260–1268. 10.1111/j.1365-2427.2004.01266.x

[ece311539-bib-0025] Macneale, K. H. , Peckarsky, B. L. , & Likens, G. E. (2005). Stable isotopes identify dispersal patterns of stonefly populations living along stream corridors. Freshwater Biology, 50(7), 1117–1130. 10.1111/j.1365-2427.2005.01387.x

[ece311539-bib-0026] Malicky, H. (1987). Anflugdistanz und Fallenfangbarkeit von Köcherfliegen (Trichoptera) bei Lichtfallen. Jahresbericht Biologische Station Lunz, 10, 140–157.

[ece311539-bib-0027] Meier, C. , Haase, P. , Rolauffs, P. , Schindehütte, K. , Schöll, F. , Sundermann, A. , & Hering, D . (2006). Methodisches Handbuch Fließgewässerbewertung zurUntersuchung und Bewertung von Fließgewässern auf der Basis desMakrozoobenthos vor dem Hintergrund der EG‐Wasserrahmenrichtlinie. http://www.fliessgewaesserbewertung.de/

[ece311539-bib-0028] Nachev, M. , Jochmann, M. A. , Walter, F. , Wolbert, J. B. , Schulte, S. M. , Schmidt, T. C. , & Sures, B. (2017). Understanding trophic interactions in host‐parasite associations using stable isotopes of carbon and nitrogen. Parasites & Vectors, 10(1), 90. 10.1186/s13071-017-2030-y 28212669 PMC5316170

[ece311539-bib-0029] Naman, S. M. , Rosenfeld, J. S. , Third, L. C. , & Richardson, J. S. (2017). Habitat‐specific production of aquatic and terrestrial invertebrate drift in small forest streams: Implications for drift‐feeding fish. Canadian Journal of Fisheries and Aquatic Sciences, 74(8), 1208–1217. 10.1139/cjfas-2016-0406

[ece311539-bib-0030] Nathan, R. , Klein, E. , Robledo‐Arnuncio, J. J. , & Revilla, E. (2012). Dispersal kernels: review. In J. Clobert , et al. (Eds.), Dispersal ecology and evolution (pp. 186–210). Oxford University Press.

[ece311539-bib-0031] Peredo Arce, A. , Hörren, T. , Schletterer, M. , & Kail, J. (2021). How far can EPTs fly? A comparison of empirical flying distances of riverine invertebrates and existing dispersal metrics. Ecological Indicators, 125, 107465. 10.1016/j.ecolind.2021.107465

[ece311539-bib-0032] Petersen, I. , Winterbottom, J. H. , Orton, S. , Friberg, N. , Hildrew, A. G. , Spiers, D. C. , & Gurney†, W. S. C. (1999). Emergence and lateral dispersal of adult Plecoptera and Trichoptera from Broadstone stream, UK. Freshwater Biology, 42(3), 401–416. 10.1046/j.1365-2427.1999.00466.x

[ece311539-bib-0033] Posit Team . (2023). RStudio: Integrated development environment for R. Posit Software, PBC. http://www.posit.co/

[ece311539-bib-0034] Prati, S. , Enß, J. , Grabner, D. S. , Huesken, A. , Feld, C. K. , Doliwa, A. , & Sures, B. (2023). Possible seasonal and diurnal modulation of Gammarus pulex (Crustacea, Amphipoda) drift by microsporidian parasites. Scientific Reports, 13(1), 9474. 10.1038/s41598-023-36630-2 37301923 PMC10257654

[ece311539-bib-0035] R Core Team . (2021). R: A language and environment for statistical computing. (Version 4.1.2). R Foundation for Statistical Computing. https://www.r‐project.org/

[ece311539-bib-0036] Radinger, J. , Kail, J. , & Wolter, C. (2014). FIDIMO—A free and open source GIS based dispersal model for riverine fish. Ecological Informatics, 24, 238–247. 10.1016/j.ecoinf.2013.06.002

[ece311539-bib-0037] Rawer‐Jost, C. , Kappus, B. , Böhmer, J. , Jansen, W. , & Rahmann, H. (1998). Upstream movements of benthic macroinvertebrates in two different types of fishways in southwestern Germany. Hydrobiologia, 391(1/3), 47–61. 10.1023/A:1003594726288

[ece311539-bib-0038] Sánchez‐Carrillo, S. , & Álvarez‐Cobelas, M. (2018). Stable isotopes as tracers in aquatic ecosystems. Environmental Reviews, 26(1), 69–81. 10.1139/er-2017-0040

[ece311539-bib-0039] Sarremejane, R. , Cid, N. , Stubbington, R. , Datry, T. , Alp, M. , Cañedo‐Argüelles, M. , Cordero‐Rivera, A. , Csabai, Z. , Gutiérrez‐Cánovas, C. , Heino, J. , Forcellini, M. , Millán, A. , Paillex, A. , Pařil, P. , Polášek, M. , Tierno de Figueroa, J. M. , Usseglio‐Polatera, P. , Zamora‐Muñoz, C. , & Bonada, N. (2020). DISPERSE, a trait database to assess the dispersal potential of European aquatic macroinvertebrates. Scientific Data, 7(1), 386. 10.1038/s41597-020-00732-7 33177529 PMC7658241

[ece311539-bib-0040] Schmidt‐Kloiber, A. , & Hering, D. (2015). ww w.freshwaterecology.info – An online tool that unifies, standardises and codifies more than 20,000 European freshwater organisms and their ecological preferences. Ecological Indicators, 53, 271–282. 10.1016/j.ecolind.2015.02.007

[ece311539-bib-0041] Schülting, L. , Feld, C. K. , & Graf, W. (2016). Effects of hydro‐ and thermopeaking on benthic macroinvertebrate drift. The Science of the Total Environment, 573, 1472–1480. 10.1016/j.scitotenv.2016.08.022 27515014

[ece311539-bib-0042] Sondermann, M. , Gies, M. , Hering, D. , Winking, C. , & Feld, C. K. (2017). Application and validation of a new approach for modelling benthic invertebrate dispersal: First colonisation of a former open sewer system. The Science of the Total Environment, 609, 875–884. 10.1016/j.scitotenv.2017.07.142 28783900

[ece311539-bib-0043] Tank, J. L. , Martí, E. , Riis, T. , Von Schiller, D. , Reisinger, A. J. , Dodds, W. K. , Whiles, M. R. , Ashkenas, L. R. , Bowden, W. B. , Collins, S. M. , Crenshaw, C. L. , Crowl, T. A. , Griffiths, N. A. , Grimm, N. B. , Hamilton, S. K. , Johnson, S. L. , McDowell, W. H. , Norman, B. M. , Rosi, E. J. , … Webster, J. R. (2018). Partitioning assimilatory nitrogen uptake in streams: an analysis of stable isotope tracer additions across continents. Ecological Monographs, 88(1), 120–138.

[ece311539-bib-0044] Tank, J. L. , Meyer, J. L. , Sanzone, D. M. , Mulholland, P. J. , Webster, J. R. , Peterson, B. J. , Wollheim, W. M. , & Leonard, N. E. (2000). Analysis of nitrogen cycling in a forest stream during autumn using a 15 N‐tracer addition. Limnology and Oceanography, 45(5), 1013–1029. 10.4319/lo.2000.45.5.1013

[ece311539-bib-0045] Tonkin, J. D. , Altermatt, F. , Finn, D. S. , Heino, J. , Olden, J. D. , Pauls, S. U. , & Lytle, D. A. (2018). The role of dispersal in river network metacommunities: Patterns, processes, and pathways. Freshwater Biology, 63(1), 141–163. 10.1111/fwb.13037

[ece311539-bib-0046] Vance, S. A. (1996). The effect of the mermithid parasite Gasteromermis sp. (Nematoda: Mermithidae) on the drift behaviour of its mayfly host, Baetis bicaudatus (Ephemeroptera: Baetidae): A trade‐off between avoiding predators and locating food. Canadian Journal of Zoology, 74(10), 1907–1913. 10.1139/z96-215

[ece311539-bib-0047] Vos, M. , Hering, D. , Gessner, M. O. , Leese, F. , Schäfer, R. B. , Tollrian, R. , Boenigk, J. , Haase, P. , Meckenstock, R. , Baikova, D. , Bayat, H. , Beermann, A. , Beisser, D. , Beszteri, B. , Birk, S. , Boden, L. , Brauer, V. , Brauns, M. , Buchner, D. , … Sures, B. (2023). The asymmetric response concept explains ecological consequences of multiple stressor exposure and release. The Science of the Total Environment, 872, 162196. 10.1016/j.scitotenv.2023.162196 36781140

[ece311539-bib-0048] Winking, C. , Lorenz, A. W. , Sures, B. , & Hering, D. (2016). Start at zero: Succession of benthic invertebrate assemblages in restored former sewage channels. Aquatic Sciences, 78(4), 683–694. 10.1007/s00027-015-0459-7

